# Tumor necrosis factor alfa and interleukin 1 alfa induced phosphorylation and degradation of inhibitory kappa B alpha are regulated by estradiol in endometrial cells

**DOI:** 10.4274/tjod.47700

**Published:** 2018-03-29

**Authors:** Sefa Arlıer, Ümit Ali Kayışlı, Aydın Arıcı

**Affiliations:** 1 University of South Florida Faculty of Medicine, Department of Obstetrics and Gynecology, Tampa, USA; 2 University of Health Sciences, Adana Numune Training and Research Hospital, Clinic of Obstetrics and Gynecology, Adana, Turkey; 3 Yale University Faculty of Medicine, Department of Obstetrics and Gynecology, Division of Reproductive Endocrinology and Infertility, New Haven, USA; 4 Anadolu Medical Center, Clinic of Reproductive Endocrinology and Infertility, İstanbul, Turkey

**Keywords:** Inhibitory kappa Bα, nuclear factor kappa B, estradiol, endometrium, tumor necrosis factor-α

## Abstract

**Objective:**

When bound to the inhibitory kappa B (IкB) protein, the transcription factor nuclear factor kappa B (NF-кB) remains inactively in the cytoplasm. Activated NF-кB upregulates the gene expression of many chemokines including monocyte chemoattractant protein-1 and interleukin (IL)-8. We hypothesized that estrogen may regulate IкB phosphorylation and degradation thus influencing NF-кB-dependent gene expression. Regulation of chemokines by estrogen is different in uterine endometrial cells when compared to ectopic endometrial cells of endometriosis.

**Materials and Methods:**

We investigated the in vivo expression of IкB in normal endometrium and in eutopic and ectopic endometrium of women with endometriosis. We then studied in cultured endometrial cells to assess the effects of estradiol on IкB and NF-кB function.

**Results:**

Normal endometrium from mid-late proliferative phase revealed the strongest IкB immunoreactivity throughout the cycle (p<0.05). When compared to paired homologous eutopic endometrium, ectopic endometrium revealed significantly less immunoreactivity for IкB (p<0.05). Moreover, estradiol induced a decrease in tumor necrosis factor-and IL-1-induced IкB phosphorylation, and also decreased the levels of active-NF-кB (p<0.05).

**Conclusion:**

Our results support the conclusion that one pathway for estradiol-mediated NF-кB inhibition occurs through the down-regulation of IкB phosphorylation. We propose that the estradiol-induced regulation of IкB and consequent reduction in active-NF-кB may affect inflammatory responses in human endometrial cells.

**PRECIS:** We have assessed that the estradiol-stimulated regulation of inhibitory kappa Bα and subsequent decrease in nucleor factor faktör kappa B affects inflammatory reactions in human endometrial cells.

## Introduction

Immunologic-endocrine interactions mediate and participate in complex physiologic processes that occur within the uterus throughout the menstrual cycle and pregnancy, and are also important to the pathophysiology of endometriosis^(1,2,3)^. One of the molecular signaling pathways that may be regulated by the endocrine system, which also participates in the regulation of inflammation, is the nuclear factor kappa B (NF-kB) signaling cascade^(4,5,6)^. NF-kB is a transcription factor that is kept in an inactive state in the cytosol while bound to the inhibitory kappa B (IkB) protein^(7,8)^. First described in B cells, NF-kB was subsequently recognized as a nuclear and cytoplasmic protein that is found in multiple cell types^(9)^. In many cells, NF-kB positively regulates the expression of a number of genes including those of cytokines, cell adhesion molecules, complement factors, anti-apoptotic factors, and immunoreactions^(10,11,12)^. The IkB protein family is composed of 35-70 kDa proteins that are localized in the cytoplasm and inhibit the activation of NF-kB. This protein family includes IkBa, IkBb, IkBg, IkB-R, B-cell leukemia-3, p105/p50, p100/52 and the Drosophila melanogaster proteins Cactus and Relish. IkBa and IkBb preferentially interact with NF-κB dimers composed of proteins p65 and p50, and regulate NF-kB function by converting the heterodimer structure to a trimer that is incapable of binding DNA^(13,14,15)^. Tumor necrosis factor-alpha (TNF-α) and interleukin (IL)-1 induce the phosphorylation and subsequent degradation of IkBα. This, in turn, results in the activation and relocation of NF-kB to the nucleus, leading to NF-kB-mediated transcription of responsive genes^(16,17)^. Ligand binding to most, if not all, of the inflammatory cytokine receptors activates intracellular signaling molecules that engender the activation of NF-kB. Activation of such signaling molecules results in a transient activation of IkB kinase (IKK) and a transient phosphorylation of IkBα (phospho-IkBα). Often, phospho-IkBα peaks 2-15 min after stimulation with the cytokine, and is followed by a rapid acceleration of IkBα degradation. Often, IkBα levels may subsequently increase in the cytosol over the following 2-6 h, in response to NF-kB-mediated upregulation of the IkB promotor^(11,13,18)^. Several proteins and molecules that activate NF-kB signaling have been described. IL-1 and TNF-α are two principal cytokines that promote IkBα degradation and NF-kB activation. Although these cytokines bind to specific receptors to activate different intracellular second messengers, downstream signals merge with the activation of the same target, namely IKK^(19,20,21)^. Estrogen influences the growth, differentiation, and function of many target cells by genomic and non-genomic pathways. Although the genomic effects of estrogen are mediated via estrogen receptors (ERs) and occur over a period of hours or days, the non-genomic effects occur within minutes^(22,23,24)^. Previous studies have shown that estrogen down-regulates the expression of many cytokines such as IL-1, TNF-α, IL-6 and regulated-upon activation, normal T-cell-expressed and secreted (RANTES), which are regulated by NF-kB in various cell types^(25,26,27)^. Previously, we have shown that estrogen inhibits monocyte chemotactic protein-1 expression in human endometrial stromal cells (ESCs)^(28)^. Moreover, in response to estrogen, chemokine-mediated regulation of endometrial cells obtained from women with endometriosis is distinct from that observed in normal endometrial cells^(29,30,31)^. An estrogen-dependent disease, endometriosis develops outside of the uterus and is characterized by a proinflammatory peritoneal environment^(32,33)^.Thus, there may be differential regulation of NFkB signaling by estrogen and by cytokines such as TNF-α and IL-l in endometriotic cells as compared with normal endometrial cells. In endometriotic cells, there appears to be synergy between the effects of E_2_ and IL-1/TNF-α, whereas these molecules appear to function antagonistically in normal endometrial cells. We hypothesized that estrogen might regulate IkBα phosphorylation and degradation *in vivo* and *in vitro* in normal endometrium and in eutopic and ectopic endometrium of women with endometriosis, thus influencing NFkB-dependent gene expression. First, we investigated the *in vivo* expression of IkBα in normal endometrium and in eutopic and ectopic endometrium of women with endometriosis. We then investigated the modulation of IkBα by E_2_ in TNF-α- and IL-1α-treated endometrial stromal and glandular cells, *in vitro*, using Western blot analysis and immunocytochemistry.

## Materials and Methods

### Tissue collection

Endometrial tissues were obtained from human uteri after hysterectomy conducted for benign diseases excluding endometrial disease, and from endometrial biopsies. Approval for this study was granted by the Human Investigation Committee of Yale University (HIC#22334) and written informed consent was obtained from each patient prior to surgery. The mean age of the patients was 36 years (range, 30-45 years).

For immunohistochemistry, normal cyclic endometrium (n=12) of women without endometriosis, and eutopic and ectopic endometrium pairs of women with endometriosis (n=6) were collected, and paraffin blocks were routinely prepared and cut at 5-7 mm. For the endometrial cells used in culture, the diagnoses of the patients were leiomyomata uteri or voluntary sterilization by tubal ligation (n=5). The day of the menstrual cycle was established from the patient’s menstrual history and was verified through histologic examination of the endometrium. The tissues were placed in Hank’s balanced salt solution and transported to the laboratory for separation and culture of endometrial stromal and glandular cells. Each experimental setup was repeated on at least three occasions using cells obtained from different patients.

### Isolation and culture of human endometrial stromal and glandular cells

Endometrial tissues were separated and conserved in a monolayer culture, as described previously^(34)^. The isolated endometrial cells were separated by filtration through a wire sieve (73 mm diameter pore, Sigma). The endometrial glands (largely undispersed) were retained by the sieve, whereas the dispersed stromal cells passed through the sieve into the filtrate. The stromal cells were plated in plastic flasks (75 cm^2^, Falcon, Franklin Lakes, NJ), maintained at 37 °C in a humidified atmosphere (5% CO_2_ in air), and allowed to replicate to confluence. Thereafter, the stromal cells were passed by standard methods of trypsinization, plated in culture dishes (100 mm diameter), and allowed to replicate to confluence. ESCs after the first passage were characterized as described previously^(34) ^and were found to contain 0-7% epithelial cells, no detectable endothelial cells, and 0.2% macrophages. Experiments were commenced 1-3 days after the cells reached confluence. The confluent cells were treated with serum-free, phenol red-free media for 24 h before treatment with test agents. Stromal cells reached confluence in 7-10 days.

Experiments with glandular cells were performed using a well-differentiated endometrial adenocarcinomacell line (Ishikawa cell) provided to us by Dr. R. Hochberg (Departmentof Obstetrics and Gynecology, Yale University, New Haven, CT)from a frozen stock. Thawed cells were maintained in T75 flasks(BD Biosciences, Franklin Lakes, NJ) until passage.The cells were treated with serum-free phenol red-free media for 24 h before treatment with test agents. Cells were treated with E_2 _(Sigma) for 3-90 min and immunocytochemistry and Western blot analysis were performed as described.

### Immunohistochemistry and immunocytochemistry

Endometrial tissue sections from normal, eutopic, and ectopic endometrium were deparaffinized and washed with phosphate buffered saline (PBS). Thereafter, sections were twice microwaved in citric acid buffer (0.1 M, pH: 6) and thoroughly rinsed in PBS. The same steps used for immunocytochemistry (described below) were followed. ESCs were grown to pre-confluence on four-chamber slides. Following treatment, the chamber slides were fixed in 4% paraformaldehyde for 20 min. After several washes with distilled water and then with PBS (pH 7.4) (three times 10 min each), endogenous peroxidase activity was quenched by 3% H_2_O_2_ (0.6 mL H_2_O_2_ and 5.4 mL methanol) for 10 min and the slides were then rinsed in PBS-tween. Slides were then incubated with rabbit anti-IkBa polyclonal antibody (Cell signaling Technology, Beverly, MA) for 60 min at room temperature. In negative control slides, normal rabbit immunoglobulin G (IgG) was used as a control instead of primary antibody. After several rinses in PBS, goat biotinylated anti-rabbit IgG (Vector Laboratories, Burlingame, CA) was applied for 30 min. After several rinses with PBS, the slides were incubated with streptavidin-peroxidase complex for 30 min (Vector Laboratories). The slides were then rinsed several times in PBS and incubated with 3-amino-9-ethyl-carbazole (Vector Laboratories) for 10 min. The slides were lightly counterstained with hematoxylin prior to permanent mounting. Immunocytochemical staining intensity was ranked between 0 (absent) to 3 (most intense). For each slide, an HSCORE value was derived by summing the percentages of cell staining at each intensity multiplied by the weighted intensity of the staining [HSCORE=Σ P*_i_*(*i*+1), where *i* is the intensity scores and P*_i_* is the corresponding percentage of the cells]. In each slide, five randomly selected areas were assessed microscopically using 50*×* magnification. Two investigators who were blinded to the treatments analyzed each slide for intensity. The averages for the scores of both investigators are presented.

### IkBa and phospho-IkBa Western blot analysis

Total protein from endometrial cells was extracted in a lysis buffer composed of 50 mM hydroxyethyl piperazineethanesulfonic, pH: 7.4; 150 mM NaCl; 10% glycerol, 1% Triton X-100, 1.5 mM MgCl_2_-6H_2_O; 1 mM EGTA; 100 mM NaF, 10 mM sodium pyrophosphate and protease inhibitors, 1 mM Na_3_VO_4_, 10 mg/mL leupeptin, 10 mg/mL aprotinin; and 4 mM phenylmethylsulfonyl fluoride. The protein concentration was determined by a detergent-compatible protein assay (Bio-Rad Laboratories, Hercules, CA). Protein lysates (20 μg) were loaded and separated using sodium dodecyl sulfate-polyacrylamide gel electrophoresis with 10% Tris-Hydrogen chloride Ready Gels (Bio-Rad Laboratories) and electroblotted onto nitrocellulose membrane (Bio-Rad Laboratories). Equal loading of proteins in each lane was confirmed by staining the membrane with Ponceau 2S (Sigma). The membrane was incubated with 5% nonfat dry milk in tris-buffered saline-tween (TBS-T) buffer (0.05% tween-20 in PBS, pH 7.4) for 1 h to reduce nonspecific binding of antibody. The membrane was probed with rabbit anti-IkBα and rabbit anti-phospho-IkBα (Ser32) antibodies (Cell Signaling Technology) overnight to quantitate total and phospho-IκBα forms. After washing with TBS-T, blots were incubated for 1 h with peroxidase labeled anti-rabbit IgG (Vector Laboratories) diluted at 1:10000. Membranes were washed with TBS-T and the immunoblots were developed using chemiluminescent kit following the manufacturer’s instructions. (NEN Life Science, Boston, MA).  The signal was normalized by dividing the arbitrary densitometry units for phospho-IkBα to the amount of total IkBα for each band. The signals were quantified by using a laser densitometer (Molecular Dynamics, Sunnyvale, CA) to analyze the autoradiographic bands.

### Preparation of nuclear extracts and the active-NF-κB assay

To quantify the amount of active NF-κB, which binds to NFkB response element sites on gene promotors, an enzyme-linked immunosorbent (ELISA) plate covered with NF-κB binding consensus sequence oligonucleotide (5’-GGGACTTTCC-3’) was used in combination with nuclear extracts from our cultured cells. Two different primary antibodies against NF-κB each recognize either an epitope on p65 or on p50 that is accessible only after dissociation of IκB from NF-κB, indicating the activation of cytoplasmic NF-κB.  An horseradish peroxidase-conjugated secondary antibody provides a colorimetric readout that is quantitated using spectrophotometry (450 nm). As a positive control for activated NF-κB, nuclear extracts from HeLA cells were used. To monitor the specificity of the assay, both wild type and mutated consensus oligonucleotides were employed in each reaction. Nuclear extracts from endometrial cells grown to confluence in 60 mm plates were obtained using a nuclear extraction kit (Active Motif, Carlsbad, CA). Briefly, cells were washed with ice-cold PBS and protease/phosphatase inhibitors, removed from the dish by scraping with a cell lifter and transferred to pre-chilled tubes. Cell suspensions were centrifuged at 4 °C for 5 min at 500 rpm. Pellets were resuspended in hypotonic buffer and incubated for 15 min on ice, detergent was added, and the cells were centrifuged at 4 °C for 30 seconds at 14.000 ×g. The pellet was resuspended in a lysis buffer and incubated for 30 min on ice on a rocking platform. The suspension was centrifuged at 4 °C for 10 min at 14.000 ×g and the supernatant (nuclear fraction) was aliquoted and frozen at -80 °C. Nuclear fractions were quantitated using a Coomassie protein assay (Pierce; Rockford, IL) as per the manufacturer’s protocol. Four micrograms of nuclear extract sample were loaded into each well and assayed according to the manufacturer’s directions (Active Motif) using a microplate reader. Quantification of the NF-κB p50 subunit was expressed as mean absorbance (λ) per sample.

### Statistical Analysis

IkBα immunocytochemistry scores and Western blot results were normally distributed as assessed using the Kolmogorov-Smirnov test. Analysis of variance (ANOVA) and post hoc Tukey test for pair-wise comparisons were used in statistical analysis. p<0.05 was considered to be significant. Statistical calculations were performed using Sigma stat for Windows, version 2.0 (Jandel Scientific Corporation, San Rafael, CA).

## Results

### Expression of IкBα in normal endometrium, and in eutopic and ectopic endometrium from women with endometriosis

**Figure 1:**
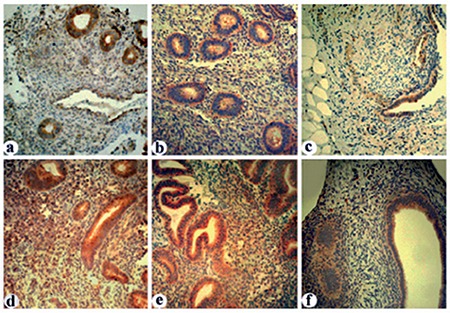
Inhibitory kappa B (IкBα) immunoreactivity in human normal (a, d), eutopic (b, e) and ectopic (c, f) endometrial tissues. IкBα immunoreactivity in proliferative (a, c) and secretory phase (d, f) tissue samples are seen. Stronger immunoreactivity in endometrial glands and stromal cells in normal endometrium are observed when compared with ectopic endometrial and stromal cells. (a-f x40)

Eutopic endometrial stromal and glandular cells from women without endometriosis express immunoreactive IkBα (Figure 1). The antibody used for immunohistochemistry recognizes both phosphorylated and unphosphorylated forms of IkBα. In normal endometrium, glandular cells reveal stronger immunoreactivity for IkBα compared with stromal cells throughout the menstrual cycle. Stronger immunoreactivity was detected in samples of mid-late proliferative endometrium compared with late secretory and early proliferative phase samples (p<0.05) (Figure 1, Table 1). When proliferative phase and secretory phase immunoreactivity for IkB were compared, the proliferative phase showed a trend for stronger immunoreactivity although this difference did not reach statistical significance. Eutopic and ectopic endometrium from women with endometriosis also revealed immunoreactivity for IkBα. When the eutopic endometrium from women with endometriosis was compared with the endometrium of women without endometriosis, no significant difference was observed in staining intensity, although eutopic endometrial cells of women with endometriosis showed a trend towards decreased immunoreactivity for IkBα (p=0.1) (Figure 1, Table 2). On the other hand, when compared with eutopic endometrium, homologous ectopic endometrium revealed significantly less immunoreactivity for IkBα (p<0.05) (Figure 1, Table 2).

### Estradiol-regulated expression of IкB in endometrial cells as assessed using immunocytochemistry

**Figure 2:**
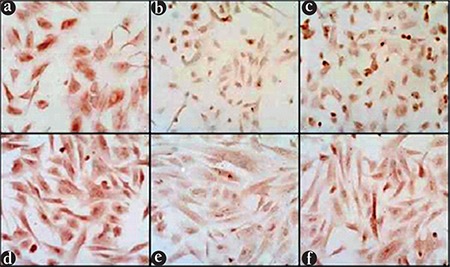
Inhibitory kappa B (IкBα) immunoreactivity in endometrial stromal cells treated with estradiol and tumor necrosis factor-alpha (TNF-α.) Endometrial stromal cells were treated for 12 min with vehicle (control) (a), TNF-α (2 ng/mL) (b), or estradiol (10^-8^ M) combined with TNF-α (c), and were immunostained for IкBα. Cells treated with estradiol combined with TNF-α showed stronger immunoreactivity for IкBα than cells treated with TNF-α alone. Endometrial stromal cells were pretreated with vehicle (control) (d, e) or estradiol (f) for 24 h prior to stimulation with TNF-α (e, f) for 15 min. Following stimulation with TNF-α cells pretreated with estradiol for 24 h (f) showed stronger immunoreactivity for IкBα than cells that were not pretreated with estradiol (e)

ESCs grown on four-chamber slides were placed in serum-free, phenol red-free media for 24 h, and were then treated for 15 min with fresh serum-free, phenol red-free media as control, with TNF-α (2 ng/mL),  or estradiol (10^-8^ M) combined with TNF-α (2 ng/mL) for 15 min. Slides were stained with rabbit anti-IkBα antibody. Cells treated with TNF-α alone showed a very weak immunoreactivity for IkBα when compared with the control (Figure 2a, b). On the other hand, cells treated with TNF-α combined with E_2_ displayed a stronger IkBα immunoreactivity than those treated with TNF-α alone (p<0.05) (Figure 2b, c).  We also compared cells maintained for 24 h in serum-free phenol red-free media for 24 h, with or without E_2_ (10^-8^ M), followed by TNF-α (2 ng/mL) treatment for an additional 15 min. TNF-α–stimulated IkBα immunoreactivity was stronger in cells pre-treated with E_2_ compared with those pre-treated with serum-free media alone (p<0.05) (Figure 2d-f).

### Regulation of IкBα expression and phosphorylation in endometrial cells as assessed using Western blot analysis

**Figure 3:**
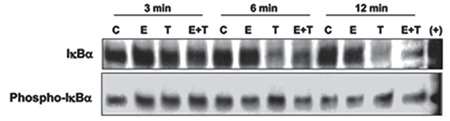
Regulation of inhibitory kappa B (IкBα) in endometrial stromal cells by estradiol and tumor necrosis factor-alpha (TNF-α). Endometrial stromal cells treated with estradiol (E_2_; 10^-8^ M), TNF-α (mg/mL) alone, or estradiol with TNF-α (E_2_+T), or vehicle (C; control) were analyzed for IкBα and its phosphorylated form following 3-12 min treatment. Estradiol treatment suppressed partially the TNF-α-induced IкBα degradation at 6 and 12 min. (+: positive control from TNF-α -induced HeLa cell extracts) *Phosphorylation of IкBα: Inhibitory kappa B alpha*

**Figure 4:**
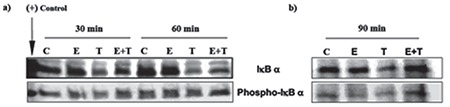
Regulation of inhibitory kappa B-alpha (IкBα) in endometrial stromal cells by estradiol and tumor necrosis factor-alpha (TNF-α). Endometrial stromal cells were treated with estradiol (E_2_); 10^-8^ M, TNF-α (T; 1 mg/mL), estradiol in addition to TNF-α (E_2_+T), or vehicle (C; control) for 30-60 min. Estradiol has a partial opposing effect on TNF-α–induced IкBα phosphorylation and degradation at both time points (a). Endometrial glandular cells were treated in a similar manner for 90 min, and similar effects were observed (b) *Phospho-IкBα: Phosphorylation of inhibitory kappa B-alpha*

We sought to understand whether the increased IkBα immunoreactivity observed in cells treated with both TNF-α and E_2_ was associated with a phosphorylation and subsequent degradation of IkBα. After  24 h of incubation with serum-free, phenol red-free media, ESCs were treated with media alone (control), E_2_ 10^-8^ M alone, TNF-α 2 ng/mL alone, or with E_2_ 10^-8^ M combined with TNF-α 2 ng/mL for 3, 6, 12, 30, and 60 min. Total protein was extracted and levels of total IкBa and phospho-IкBa were measured using Western blot analysis. Control and E_2_-treated cells showed similar levels of IkBα throughout the treatment period. On the other hand, treatment with TNF-α resulted in a time-dependent decrease in IkBα levels compared with the control. Moreover, this treatment caused a time-dependent increase in phospho-IkBα levels with a peak between 6 and 12 min of treatment. Meanwhile, E_2_ combined with TNF-α treatment showed markedly higher levels of IkBα when compared with TNF-α alone (Figure 3). When groups were compared in terms of phospho-IkBα levels, control and E_2_-treated cells revealed the lowest levels of phospho-IkBα throughout the treatment periods. However, in cells treated with TNF-α, co-treatment with E_2_ induced higher IkBα levels and lower phospho-IkBα levels during the first 12 minutes of treatments (p<0.05) (Figure 3). Following 60 min of treatment, IkBα levels were still higher in cells co-treated with E_2_ compared with cells treated with TNF-α alone (Figure 4a). Interestingly, in glandular cells, longer treatment with E_2_ with TNF-α (90 min) resulted in a significantly higher level of IkBα compared with other treatments, including the control group (Figure 4b). Glandular cells treated with E_2_ plus TNF-α demonstrated higher phospho-IkBα levels when compared with cells treated with TNF-α alone (p<0.05). 

**Figure 5:**
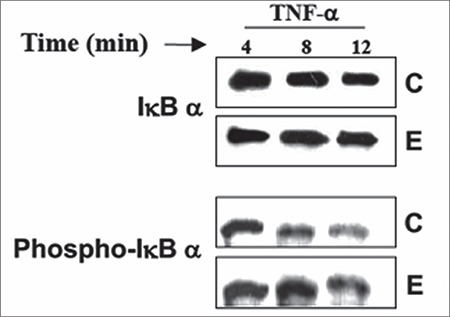
Regulation of inhibitory kappa B alpha (IкBα) in endometrial glandular cells by estradiol and tumor necrosis factor-alpha (TNF-α). Endometrial glandular cells were pre-treated with estradiol (E_2_); 10^-8^ M, or vehicle (C; control) for 24 h prior to treatment with TNF-α (1 mg/mL) for 4-12 min. E_2_ pre-treatment inhibited IкBα degradation compared with control *Phospho-IкBα: Phosphorylation of inhibitory kappa B-alpha, TNF: Tumor necrosis factor-alpha*

**Figure 6:**
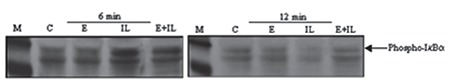
Regulation of inhibitory kappa B (IкBα) in endometrial stromal cells by estradiol and interleukin (IL)-1α. Endometrial stromal cells were treated for 6 and 12 min with estradiol (E_2_); 10^-8^ M, IL-1α (IL; E_2_ ng/mL), estradiol with IL-1к (E_2_+IL), or vehicle (C; control) and were analyzed for phospho-IкBα. Estradiol treatment suppressed IL-1α-induced IкBα degradation at 6 and 12 min *Phospho-IкBα: Phosphorylation of inhibitory kappa B-alpha, IL: Interleukin*

As observed using immunoblotting, the effect of E_2_ on IkBα was more pronounced when glandular cells were pre-treated with E_2_ for 24 h prior to TNF-α treatment (Figure 5). To determine whether the effect of E_2_ on IkBα phosphorylation was specific to the TNF-α signaling cascade, we also explored the effect of estrogen on IL-1α-mediated activation of NF-kB. Cells were treated with E_2_ (10^-8^ M), IL-1α (2 ng/mL), E_2_ plus IL-1α, or vehicle alone (control). E_2_ induced lower phospho-IkBα and higher IkBα levels in IL-1α-treated cells as compared with cells treated with IL-1α alone (Figure 6).

### Regulation of TNF-α– and IL-1α-induced activation of NF-кB by E_2_ as assessed using an NF-кB binding assay

**Figure 7:**
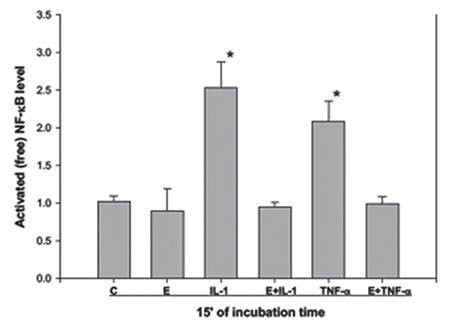
Regulation of active nuclear factor kappa B level in endometrial stromal cells by estradiol. The amount of activated NF-кB in endometrial stromal cells after 15 min of treatment with estradiol (E_2_); 10^-8^ M, interleukin (IL)-1a (IL-1; 2 ng/mL) and E_2_+IL-1 (10^-8^ M and 2 ng/mL), tumor necrosis factor-alpha (TNF-a) (TNF; 2 ng/mL) and E_2_+TNF (10^-8^ M and 2 ng/mL) were compared with control cells. Experiments were repeated on three occasions with similar results and a representative graph from one experiment is presented *NF-кB: Nuclear factor kappa B, IL: Interleukin, TNF-α: Tumor necrosis factor-alpha*

To understand whether the TNF-α– and IL-1α-induced IkBα levels in E_2_-treated cells was associated with a decrease in free NF-kB, ESCs were treated with serum-free, phenol red-free media as control, and with E_2_ (10^-8 ^M) alone, TNF-α (2 ng/mL) alone, IL-1α (2 ng/mL) alone, E_2_ combined with TNF-α or IL-1α for 15 min. Free NF-kB levels in control cells and E_2_-treated cells were lower than those in TNF-α– and IL-1α-treated cells. On the other hand, E_2_ decreased the TNF-α– and IL-1α–induced free NF-kB levels as compared with cells treated with TNF-α alone or IL-lα alone (Figure 7).

## Discussion

Steroid hormones classically bind to cognate nuclear receptors to regulate target gene expression^(35)^. Estrogen takes part in cell and tissue regulation at many stages of human life. In addition to the reproductive tract of women, other systems such as the skeletal and nervous systems are important targets for estrogen action^(36,37)^. Estrogen mainly affects cells through the genomic pathway^(38)^. Estrogen actions may also result from non-genomic activity, possibly related to the cell type, receptor type, and the presence of intracellular co-factors that may interact with typical or atypical ERs. Non-genomic effects occur within minutes and appear to include cell membrane-dependent signaling mechanisms such as the nitric oxide cascade, stimulation of p38-mitogen-activated protein kinase, or phosphorylation of protein kinase B, among others^(39,40,41,42)^. In contrast, long-term effects of estrogen, namely genomic effects, arise over hours or longer and are directed in part by DNA estrogen response elements^(43)^. Some biologic processes can also play a role in both genomic and nongenomic pathways. A previous study showed that the lipopolysaccharide-stimulated activation of NF-kB was reduced by cell-impermeable E_2_–bovine serum albumin in mouse bone marrow-derived macrophage cultures in both genomic and nongenomic pathways^(44)^. Eutopic and ectopic endometrium undergoes cycle-dependent changes predominantly controlled by estrogen and progesterone in their implantation site^(45,46,47)^. The present study is focused on the anti-inflammatory effects of estrogen, assessing IkBα phosphorylation and NF-kB activation in endometrial and endometriotic cells. *In vitro* and *in vivo* studies indicate that NF-kB–mediated gene transcription stimulates inflammation, invasion, angiogenesis, and cell proliferation, and reduces apoptosis of endometriotic cells. Excessive activation of NF-kB has been confirmed in endometriotic implants and peritoneal macrophages of patients with endometriosis^(48,49)^. In inflammatory tissue, an increase in TNF-α is often the first step in the cascade, followed by increases in the expression of various chemokines and the recruitment of leukocytes^(27,50,51,52,53)^. Previous studies have shown that, when bound to their receptors, TNF-α and IL-1 increase IkBα phosphorylation, degradation, and eventually NF-kB activation, which results in increased inflammatory cells and expression of several inflammatory cytokines and chemokines^(27,54,55)^. Our findings suggest that E_2_ may reduce phospho-IkBα and therefore decrease its degradation in endometrial cells. In this way, estrogen may block NF-kB transport into the nucleus and attenuate the inflammatory response. To our knowledge, this is the first study to report IkBα regulation by estrogen in endometrial stromal and glandular cells. It is possible that this increase arises from effects on the transcriptional or translational machinery, because a previous study has shown that E_2_ has a down-regulatory effect on IkBα at the mRNA level in phorbol ester-induced HeLa cells^(56)^. Alternatively, a previous study performed using MCF-7 cells suggested that this increase was related to the increase of p105 protein level^(57)^. On the other hand, another research group showed that estrogen treatment decreased liver IkB mRNA and protein expression and also increased ethanol-induced liver NF-kB levels and TNF-α expression^(58)^. These disparate findings are likely to be related to the cell-specific effects of estrogen and merit further analysis. Several cytokines participate in NF-kB activation. In addition to TNF-α, IL-1α also regulates IkBα levels in the cytosol. The similar effects on IkBα levels by E_2_ co-treatment with TNF-α and with IL-1α, compared with treatments with TNF-α or IL-1α alone, indicate that the effect of E_2_ is not specific for the TNF-α signaling cascade. IL-1α initiates an alternate cascade for IkBα-related NF-kB activation to that of TNF-α. Furthermore, because both signaling pathways merge on IKK activation, the effect of estrogen may be on IKK activation or on subsequent steps. Bulun et al.^(59) ^studied NF-kBα and IkBα expression in human fetal membranes and decidua at preterm and term gestation. The authors observed a marked increase in the nuclear localization of p65 and in the IkBα immunoreactivity in tissues obtained at term compared with tissues delivered preterm, suggesting a role for p65 in the regulation of parturition-related gene transcription in the decidua^(59)^. Our *in vivo* results show an increase in IkBα levels from early proliferative to the late proliferative phase, and suggest direct or indirect estrogenic regulation of IkBα in human endometrial cells. On the other hand, persistently low levels of IkBα immunoreactivity in ectopic endometrial cells are likely to be related to the increased local inflammation observed in endometriosis and may contribute to the increased inflammatory cytokine levels in the peritoneal cavity of women with endometriosis^(60,61)^. Endometriosis is an estrogen-dependent disease and implants of endometriosis have sufficient enzymes for the local production of estrogen^(54,59,62,63,64)^. The low levels of IkBα in ectopic endometrial cells suggest that the signaling effects of estrogen on IkBα may function similarly to those observed in eutopic endometrium. It seems that there is a lack of the inhibitory effect of E_2_ on cytokine-induced IkBα phosphorylation in ectopic endometrium. Supporting this hypothesis, a recent study has shown that E_2_ increases phospho-IkB levels, and more interestingly, induces higher IL-8 levels in endometriotic cells when compared with eutopic endometrium^(65)^. Similarly, Akoum et al.^(66)^  showed that E_2_ and IL-1b had synergistic effects on the expression of RANTES, revealing that E_2_ enhanced the mRNA stability of RANTES, and IL-1b increased its transcription. A recent study reported the expressions of IkBα, IkBβ, and p50 in human endometrial cells throughout the menstrual cycle^(67)^. Expression of these inhibitory proteins decreased significantly during the mid-secretory phase of the cycle. The study detected maximal immunoreactivity for IkBα during the late proliferative phase, consistent with our findings. Another study showed an increase in IkBα mRNA levels in the pre-menstrual endometrium, suggesting activation of NF-kB during this phase or alternate regulation of IkBα expression^(68)^. Our results support the findings of this study because activation of NF-kB requires IkBα phosphorylation and degradation, low levels of IkBα protein would stimulate high level of IkBα mRNA during the pre-menstrual phase to replenish degraded IkB protein. One reason for the inhibitory effect of estrogen on chemokine expression may be related to decreased IkBα degradation. As a consequence, estrogen may decrease the amount of free-NF-kB in the cytosol, and therefore decrease the level of activation. Recently, we showed that the presence of ligand ERs suppressed free-NF-kB subunits (both p65 and p50) binding to NF-kB response element,^(26)^ suggesting a second mechanism for estrogen-dependent inhibition of NF-kB-mediated gene activation. ERs in ESCs inhibited DNA binding of p50 and p65 subunits of NF-kB. Also, NF-kB activation significantly reduced estrogen responsiveness of ER-alpha–transfected ESCs, but p50 did not impair ER-alpha DNA binding, suggesting possible indirect mechanisms for this type of interaction^(26)^.

### Study Limitations

There were some limitations in the present study. This study presented a limitation with regard to experimental circumstances. These results also need to be assessed under *in vivo* conditions.

## Conclusion

Our results support the hypothesis that E_2_ inhibits NFkB activation through the down-regulation of IkBα phosphorylation and consequent reduction of free NF-kB in the cytosol. These results demonstrate that the regulation of IkBα by E_2_ may regulate the inflammatory response in eutopic and ectopic endometrial cells. Our *in vivo* and *in vitro* findings suggest that this effect of estrogen on IkBα may not be optimal in ectopic endometrium, which may be an important factor in the pathogenesis of endometriosis.

## Figures and Tables

**Table 1:**
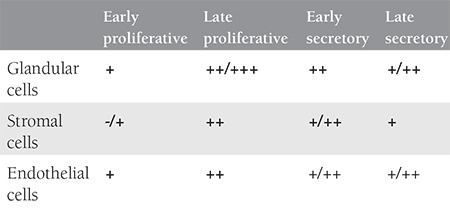
Inhibitory kappa B immunoreactivity in various cell types of human endometrium throughout the cycle. Early proliferative (n=2), late proliferative (n=4), early secretory (n=4) and late secretory (n=2)

**Table 2:**
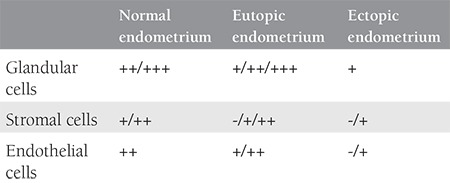
Inhibitory kappa B immunoreactivity in various cell types of normal, eutopic and ectopic endometrium. Menstrual cycle matched normal endometrium (n=6), eutopic and ectopic pairs of endometriotic endometrium samples (n=6)
